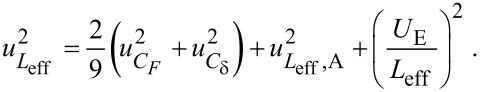# A simple method for the determination of qPlus sensor spring constants

**DOI:** 10.3762/bjnano.6.177

**Published:** 2015-08-14

**Authors:** John Melcher, Julian Stirling, Gordon A Shaw

**Affiliations:** 1 National Institute of Standards and Technology, Gaithersburg, MD 20899, USA

**Keywords:** atomic force microscopy, calibration, non-contact atomic force microscopy, qPlus

## Abstract

qPlus sensors are widely used to measure forces at the atomic scale, however, confidence in these measurements is limited by inconsistent reports of the spring constant of the sensor and complications from finite tip heights. Here we combine a numerical investigation of the force reconstruction with an experimental characterization of the flexural mechanics of the qPlus sensor. Numerical studies reveal significant errors in reconstructed force for tip heights exceeding 400 μm or one sixth of the cantilever length. Experimental results with a calibrated nanoindenter reveal excellent agreement with an Euler–Bernoulli beam model for the sensor. Prior to the attachment of a tip, measured spring constants of 1902 ± 29 N/m are found to be in agreement with theoretical predictions for the geometry and material properties of the sensor once a peaked ridge in the beam cross section is included. We further develop a correction necessary to adjust the spring constant for the size and placement of the tip.

## Introduction

Non-contact-atomic force microscopy (ncAFM) has paved new inroads to the measurement of nanometer-scale properties that were previously inaccessible. By allowing the atomic-scale imaging of surfaces from insulators to conductors, the technique opens up a broad materials spectrum to the possibility of atomic-scale analysis. This new capability has led to imaging with sub-atomic resolution [1], and chemical identification of surface atoms [2] and molecules [3], as well as dynamic force spectroscopy in a wet chemical environment [4]. However, a direct comparison between theory and experiment requires that an absolute, quantitative framework for the measurement is established, as illustrated by a recent work in single dimer manipulation [5].

In recent years, quartz tuning fork sensors have emerged as an attractive alternative to traditional silicon microcantilevers for ncAFM. The stiff spring constant of the tuning fork enables precise control over the tip–sample separation at short stand-off distances despite relatively large van der Waals interactions. Moreover, the mass production of tuning forks for timing applications has provided highly-stable frequencies with self-sensing and self-actuating electromechanical properties all at low cost [6]. Tuning fork sensors were originally used as a traditional, dual-tine oscillator. This evolved into the more widely used qPlus configuration where the sensing tine oscillates and the second tine is immobilized [7–8]. If the tines are well balanced, the former benefits from high quality factors due to low inertial coupling with the stage. The later simplifies the modeling and calibration effort for quantitative force measurements [9]. With careful design and experimentation it is possible for qPlus sensors [10], and other sensors with cantilevered geometries [11], to reach quality factors in excess of 10^6^ without inertial cancelling.

Several methods have been developed to reconstruct the tip–sample interaction force from the frequency shift of an oscillating tip in ncAFM [12–17]. This analysis requires four separate experimental inputs: the frequency shift Δω as a probe interacts with a surface relative to the unperturbed resonant frequency ω_0_, the sensor oscillation amplitude *a*, which is held constant, and *z* is the distance of nearest approach between a surface and the oscillating probe tip, and the spring constant *k*. The reconstructed tip–sample force 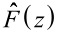 is given by [17]:

[1]
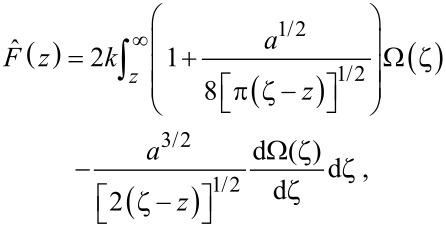


where Ω(*z*) = Δω(*z*)/ω_0_. The reconstruction requires that the *z*-separation between the tip and sample is varied while the frequency shift is monitored. Force reconstruction using other methods, such as the matrix method [15], also use the same input parameters. For a more in-depth comparison of force reconstruction methods see [18].

To extract meaningful forces from Equation 1, the input parameters must be calibrated. The accepted method for trustworthy calibrations is to establish an unbroken chain of comparisons to a internationally recognized standard, that is, to establish traceability to a primary standard. Traceability ensures that all measurements are identically scaled, allowing for consistent comparison between theory and independent measurements.

The largest source of uncertainty in ncAFM measurements currently comes from the calibration of the spring constant of the sensor. Several traceable calibration methods have been developed for micro-fabricated silicon cantilevers [19–24]. However, despite several attempts to determine spring constants for sensors based on quartz tuning forks [25–29], no comprehensive framework yet exists due to inconsistencies between results from different methods.

**Figure 1:**
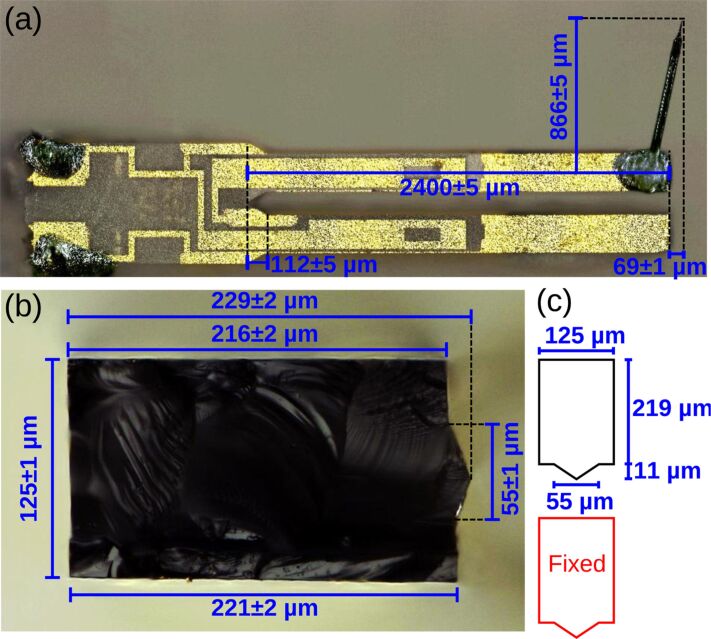
Optical images of a commercial qPlus sensor [8]. (a) Plane-view image consisting of an E158 quartz tuning fork (Micro Crystal, Switzerland) that is glued to a ceramic mount such that one tine is immobilized. A tungsten wire tip is attached to the distal end of the unconstrained tine. The tip height (measured from the center of the beam) is *h* = 866 ± 5 μm. The tip also extends the length of the cantilever by 69 ± 1 μm. (b) Optical image of a cleaved E158 tine showing the non-rectangular cross-section (image is a composite of nine images at slightly different focal lengths). (c) Model schematic of the tine cross-section.

qPlus sensors are stiff compared to traditional microcantilever sensors and present their own set of spring constant calibration challenges. A common approach has been to estimate the spring constant from plane view geometry and the Young’s modulus of the appropriate crystallographic orientation. In this case, the qPlus sensor is treated as a uniform, rectangular cantilever and the spring constant is predicted from Euler–Bernoulli beam theory [1,7]. However, qPlus sensors violate several of the assumptions inherent in this approach (see Figure 1). In particular, the cross-section of the tine is not rectangular, but rather includes a peaked ridge resulting from anisotropy in the crystal etching process [30]. The assumption of axial uniformity is violated by the chamfered edge at the base of the tine, and the assumption of base rigidity has been questioned [25]. The attachment of a tip can alter the length of the cantilever, introduce parasitic tip motion [31], and, in extreme cases, introduce additional vibratory modes [32–33].

In what follows, we develop a rigorous mathematical model for the qPlus sensor with a finite tip. The effect of the parasitic tip motion on the reconstructed interaction force is examined quantitatively from the perspective of two-dimensional grid spectroscopy. In addition, we use a traceable nanoindenter to accurately characterize the flexural mechanics of the qPlus sensor. The experimental results provide validation of a theoretical model that can be used to predict the spring constant of the qPlus sensor.

## Results

In this section we develop a model for the qPlus sensor with a finite tip that is subjected to a tip–sample interaction potential. The effect of the tip height and resulting parasitic tip rotation are carefully considered in terms of the error in the reconstructed tip–sample force.

### Modeling the qPlus sensor dynamics

**Figure 2:**
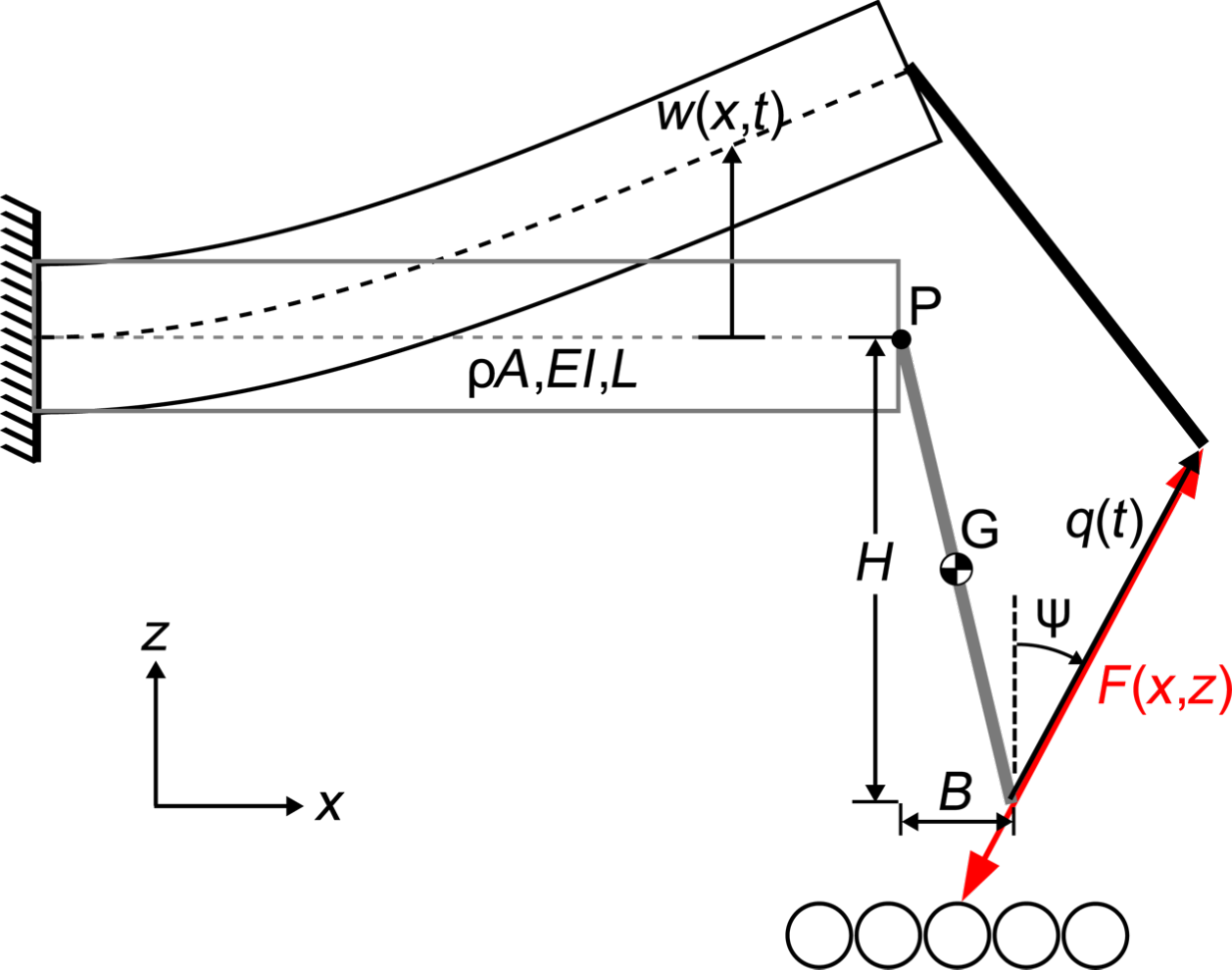
Model schematic for the qPlus sensor. The tine is a uniform beam with linear mass density μ, flexural rigidity *EI* and length *L*. The tip is attached to the free-end of the tine at point P corresponding to the neutral axis of the tine. The tip is modeled as a rigid, slender rod with a height *H* and axial offset *B*. Point G marks the center of mass of the tip.

Figure 2 provides a model schematic of the qPlus sensor. The unconstrained tine is treated as a uniform cantilever the cross-section of which is rectangular with a triangular ridge (see Figure 1). The 50 μm tungsten wire tip is modeled as a rigid, slender rod extending from the center of the distal end of the beam with height *H* and axial offset *B*. Numerical values of the modeling parameters are listed in Table 1.

**Table 1:** qPlus model parameters.

beam properties	

linear mass density (μ)	2650 kg/m^3^
Young’s modulus (*E*)	78.6 GPa
second moment of area (*I*)	1.17 × 10^−16^ m^4^
flexural rigidity (*EI*)	9.20 μN·m^2^
length (*L*)	2.4 mm

tip properties	

mass density (ρ)	19 250 kg/m^3^
diameter (*D*)	50 μm
height (*H*)	variable
axial offset (*B*)	variable

The schematic in Figure 2 highlights a kinematic property of a bending cantilever combined with a finite tip length [31]. The bending of the cantilever results in a transverse deflection of the free end. Additionally, bending causes the cross section of the beam to rotate. This rotation, coupled to a finite tip, results in an unwanted lateral displacement of the distal end of the tip. The resulting displacement of the tip occurs at an angle ψ from the normal, which depends strongly on the length of the tip. Consequently, the *z*-displacement of the sensor by the scanning stage, which is assumed to be transverse to the cantilever axis, and the tip–displacement are no longer collinear.

The dynamics of the qPlus sensor subjected to an interaction potential *V*(*x*,*z*) can be modeled with classic Euler–Bernoulli beam theory. Let *w*(*x*,*t*) denote the transverse deflection of the neutral axis (in this case, the midplane) of the cantilever. The kinetic and potential energy of the QTF sensor, respectively, are given by

[2]
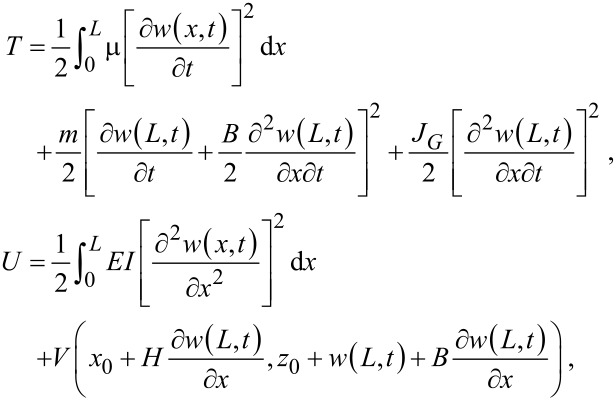


where the mass and moment of inertia of the tip are *m* = ρπ*D*^2^/4 and *J*_G_ = *m*(*H*^2^ + *B*^2^)/12. Application of Hamilton’s principle to Equation 2 establishes the governing partial differential equation (PDE) with the appropriate boundary conditions:

[3]
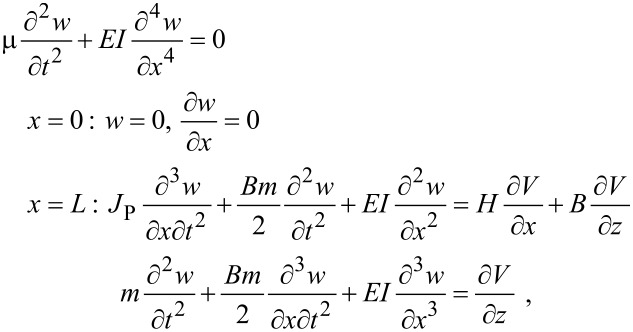


where *J*_P_ = *m* (*H*^2^ + *B*^2^)/3 is the moment of inertia of the tip about point P.

The unperturbed eigenmodes and eigenfrequencies of the qPlus sensor are solved for in the traditional manner by setting *V*(*x*,*z*) = 0 and substituting *w*(*x*,*t*) = Φ(*x*)*e^i^*^ω^*^t^* into Equation 3, where Φ(*x*) is the eigenfunction (See Appendix section). Here we limit our discussion to the fundamental eigenmode of the beam as it is most relevant to ncAFM. For convenience, let *X* = *x*/*L*. The tip-displacement angle is given by

[4]
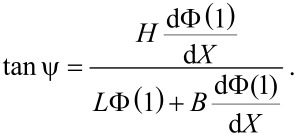


**Figure 3:**
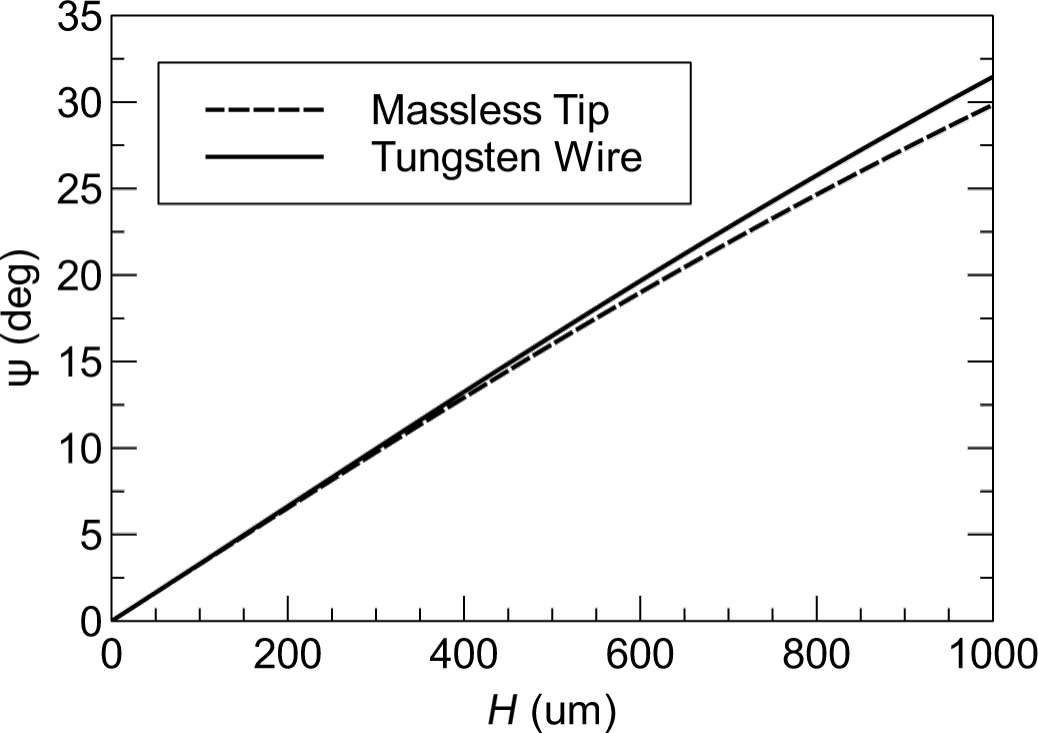
Tip displacement angle ψ vs tip height *H* calculated for the tungsten wire tip described in Table 1 and a hypothetical massless tip. *B* = 0 for both cases. The agreement between the two curves shows that ψ primarily depends on the tip height.

Figure 3 shows ψ vs *H*/*L* for *B* = 0. Results are calculated for the 50 μm tungsten wire tip and a hypothetical massless tip. ψ is shown to have strong geometric dependence on *H*/*L* in Equation 4, a weak dependence on *B*/*L*, and a weak implicit dependence on the mass and rotational inertia of the tip.

The governing PDE (Equation 3) can be reduced to an ordinary differential equation (ODE) with a single degree of freedom by approximating the motion of the beam with a single eigenmode Φ(*x*). The reduction of Equation 3 can be accomplished with a Galerkin discretization process (see Appendix section). Alternatively, one can follow the approach outlined in [34] and set *U* = *kq*^2^/2 + *V* and 
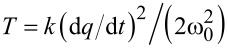
, where *k* and ω_0_ are the spring constant and unperturbed resonant frequency of the oscillator, respectively, and *q* is the coordinate for the tip displacement (See Figure 2). Following either approach, it is possible to show

[5]
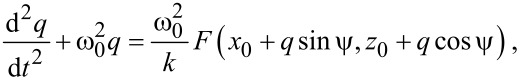


where *F*(*x*,*z*) is the derivative of *V*(*x*,*z*) in the direction of the tip displacement:

[6]
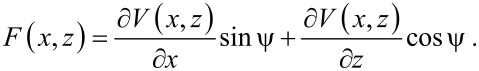


The spring constant is *k* = *k_z_* cos^2^ψ, where:

[7]
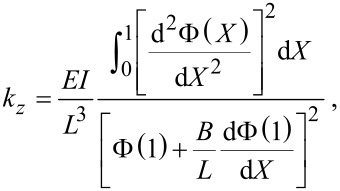


is the effective spring constant in the *z*-direction. Note that Equation 7 represents the exact solution for the spring constant of the fundamental eigenmode according to Euler–Bernoulli theory [34]. A static approximation for *k_z_*, which relates the transverse tip deflection to a static point load applied at the tip, is given by

[8]
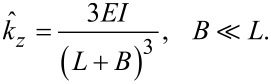


**Figure 4:**
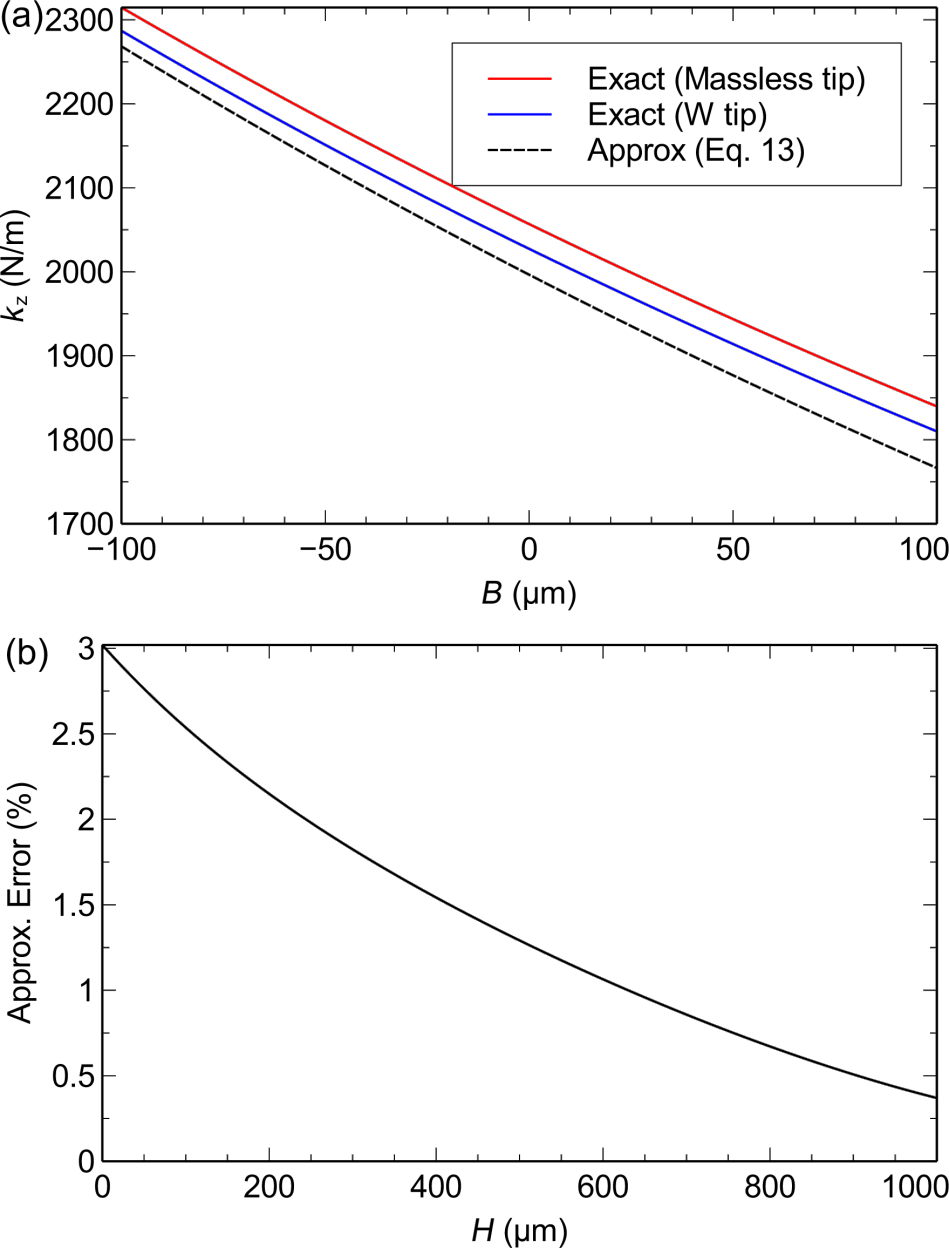
Theoretical prediction of the spring constant *k_z_*. (a) *k_z_* vs axial tip offset *B*. The spring constant is calculated with the exact expression (Equation 7) for a massless tip and for the 50 μm tungsten wire tip with *H* = 400 μm and compared to the approximate expression 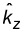 (Equation 8). (b) Approximation error 
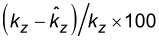
 vs *H* for *B* = 0.

Figure 4 shows the exact expression for spring constant *k_z_* according to Euler–Bernoulli theory (Equation 7) alongside the approximation 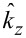 (Equation 8). The spring constant is calculated for both a massless tip and a 50 μm tungsten wire tip with *H* = 400 μm. For the massless tip, the true spring constant is about 3% stiffer than the approximation. The addition of the tip mass actually shifts the true spring constant closer to the approximate value. For a 400 μm tip height, *k_z_* is only 1.5% stiffer than 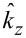 and for tip heights greater than 632 μm, the approximation deviates by less than 1%. In the following section, we neglect the error in the approximation, however, the small correction factor can be estimated from the theoretical model if desired.

### Modeling dynamic force spectroscopy

Ultimately, the goal of the modeling and calibration effort in ncAFM is to provide a quantitative measurement of the tip–sample force and/or potential. Here we will assume that the parasitic tip motion is neglected in the model and study the resulting error in the reconstructed force. It is instructive to formulate the problem from the perspective of grid spectroscopy [35], where the tip–sample force is reconstructed for a grid of points in the *xz*-plane. We study the effect of the parasitic tip motion first computing the frequency shift according to the model, and second, reconstructing the force from the Sader–Jarvis formula.

First, let us consider frequency shift, which is calculated by applying a standard perturbation approach [14,17] to Equation 5:

[9]
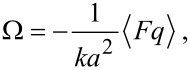


where *a* is full amplitude of the tip displacement *q*(*t*). A widely accepted method [31] for determining the oscillation amplitude in ncAFM employs a calibrated *z*-scanner and takes advantage of the large-amplitude frequency shift approximation [14]. However, this method measures only the *z*-component of the amplitude *a_z_* = *a*cosψ. Rearranging Equation 9, we find

[10]
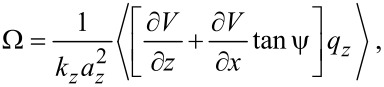


where *q_z_* = *q*cosψ is the *z*-component of the tip displacement. Thus, if only the *z*-component of the tip-displacement is measured, force measurements require knowledge of *k_z_*. However, this approach does result in a systematic error from the tanψ term in Equation 10. For large tip heights the error is significant.

To quantify the error in the reconstructed force caused by the parasitic tip motion, we introduce a model for the tip–sample interaction given by the Morse potential for a pair of silicon atoms:

[11]
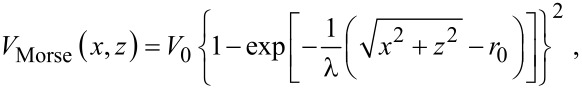


where 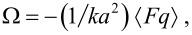 is distance between the atoms, and *V*_0_ = 3.643 × 10^−19^ J, *r*_0_ = 235.7 pm, λ = 100 pm are taken from [36]. Using Equation 10 and Equation 11, Ω(*x*,*z*) is computed for a grid of points in the *xz*-plane for an oscillation amplitude *a_z_* = 100 pm. The computation is repeated for tip heights *H* = 0, 200, 400, 600, 800 and 1000 μm, which correspond to ψ = 0, 6.5°, 13°, 19°, 25° and 30°, respectively. Substituting *a_z_* for *a*, *k_z_* for *k*, into the Sader–Jarvis formula (Equation 1) allows the tip-sample force to be reconstructed for the two-dimensional grid Ω(*x*,*z*).

**Figure 5:**
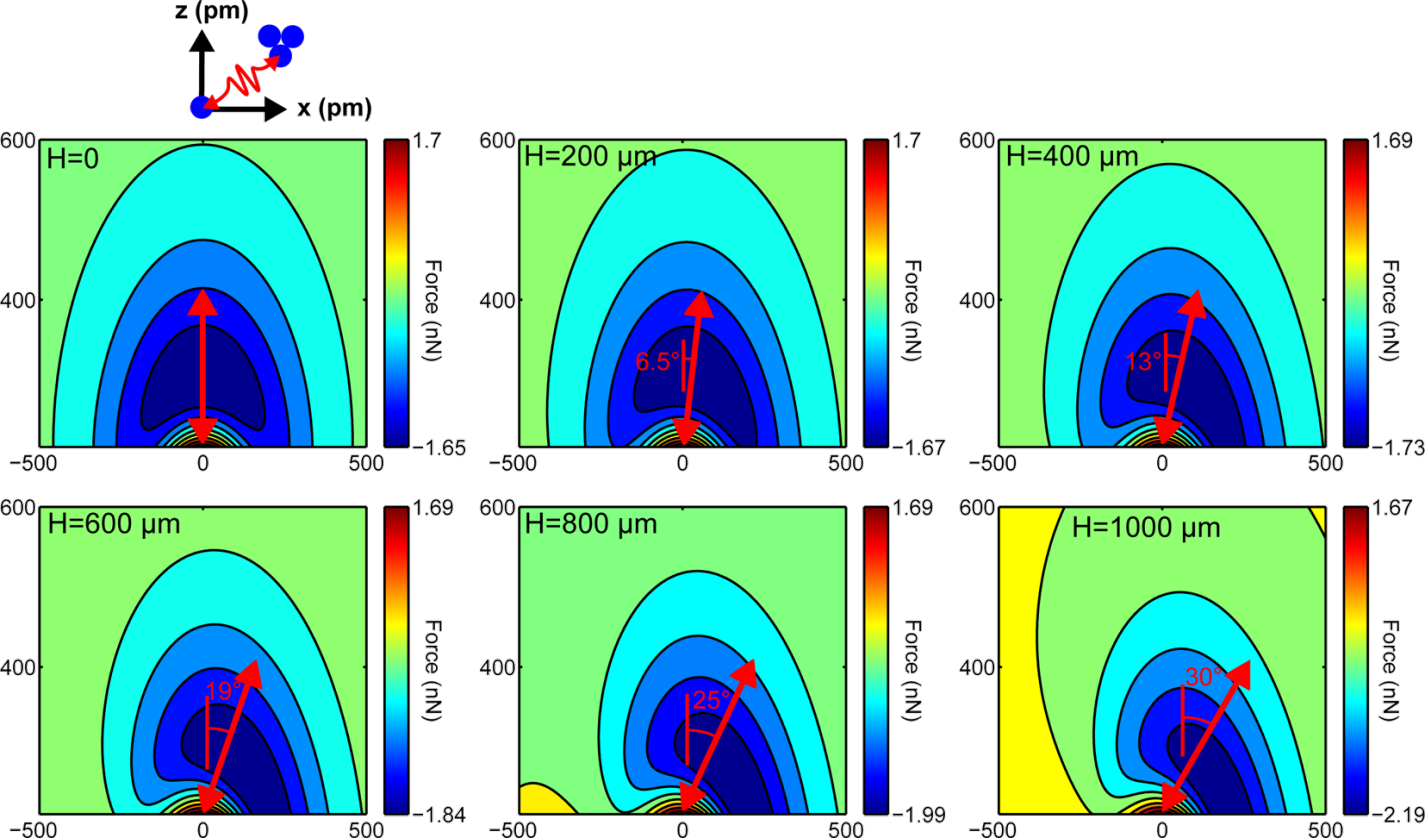
Reconstructed grid spectroscopy images for Si–Si Morse potential with varying tip heights. Red arrows are drawn to scale to indicate the direction of the tip motion. The extrema of the scale bar show the error in the reconstructed force.

Figure 5 shows two-dimensional grid spectroscopy images reconstructed from the Sader–Jarvis formula. For zero tip height, the image faithfully reconstructs the interaction force. However, for non-zero tip heights, the parasitic tip motion contributes an error to the reconstructed force. Most notably, the parasitic tip motion causes an overall distortion of the image by the angle ψ. Additionally, there is an error in the magnitude of the force, which can be quantified by the force minimum. For increasing tip heights, the reconstructed force minimum is −1.65, −1.67, −1.73, −1.84, −1.99, and −2.19 nN, respectively. Thus, for a tip height of 1000 μm, the error in the reconstructed force minimum is nearly 35%.

We remark here that it is possible, with accurate knowledge of ψ, to eliminate the error caused by the parasitic tip motion by changing the integration of Ω(*x*,*z*) in the Sader–Jarvis formula to be collinear with the tip displacement. However, this approach would be fairly onerous for most experimental setups where typically only a single frequency vs *z* curve is acquired, rather than an entire grid. Thus, the preferred method is to limit the tip height to approximately less than 400 μm.

### Mechanical characterization of qPlus sensors with nanoindentation

In this section we characterize the flexural mechanics of qPlus sensors using a nanoindentation method. The nanoindenter, which is calibrated with traceability to the International System of Units (SI) [19,21,37], measures a force vs displacement curve by pressing a sharp indenter tip into the qPlus sensor surface at a known axial distance from the distal edge of the tine. From the indentation curve, a stiffness *k*_I_ is inferred, taking care to remove the machine compliance and contact compliance by performing additional measurements at the base of the sensor. Applying this method at two or more distinct locations along the axis of the tine determines the flexural rigidity *EI* and effective cantilever length *L*_eff_ of the qPlus sensor. Moreover, the indentation data provides validation of Bernoulli–Euler beam theory with fixed-free boundary conditions to model the flexural mechanics of qPlus sensors.

Let *k*_I_(*b*) denote the force gradient measured by the nanoindenter at an offset *b* from the distal edge of the tine (positive in the +*x* direction pointing away from the base of the cantilever). For a uniform cantilever beam, the Euler–Bernoulli model predicts the following relationship:

[12]
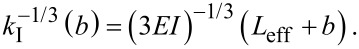


Measuring *k*_I_ for a range of offsets allows *EI* and *L*_eff_ to be determined from a linear least-squares fit regression. Note that *L*_eff_ differs slightly from the geometric length due to the non-ideal boundary conditions at the fixed end of the tine. With knowledge of *EI*, *L*_eff_ and the tip offset *B*, the spring constant *k_z_* can be determined.

**Figure 6:**
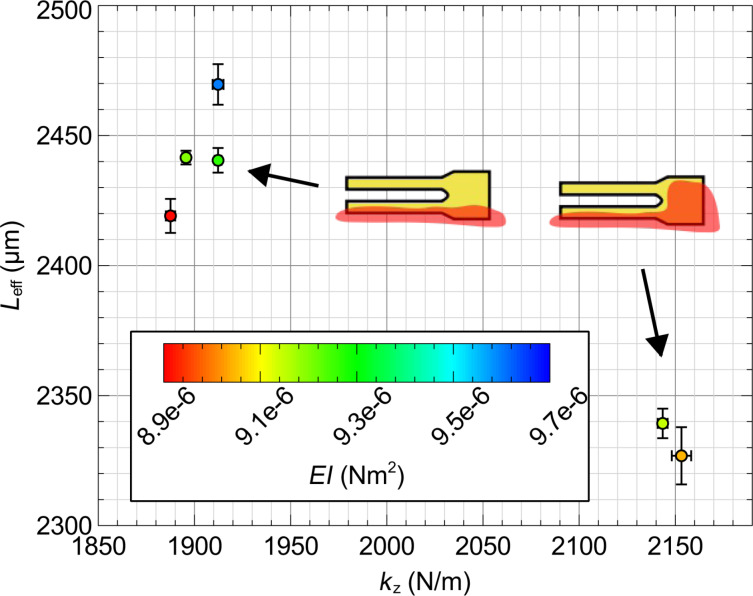
Dependence of the spring constant *k_z_* on the effective length of the cantilever *L*_eff_. Two distinct values of *L*_eff_ and corresponding *k_z_* are observed depending on the mounting of the tine. The flexural rigidity *EI* is unaffected by the mounting as expected.

The qPlus sensors tested were custom-built. E158 tuning forks were attached to ceramic substrates obtained from Oxford Instruments using Torr Seal epoxy [8]. Two gluing configurations were tested. In the first configuration, only the bottom tine is glued to the substrate, while in the second the base of the tuning fork is also glued to the substrate (cf. Figure 6). The indentations were performed with a pre-load of 1 mN and maximum load of 1.9 mN. Measurements were acquired along the axis of the tine and additionally at the base of the sensor in order to remove the contact stiffness and machine compliance from the spring constant prediction. To avoid interference with the indenter tip, tips were not attached to the tine.

**Figure 7:**
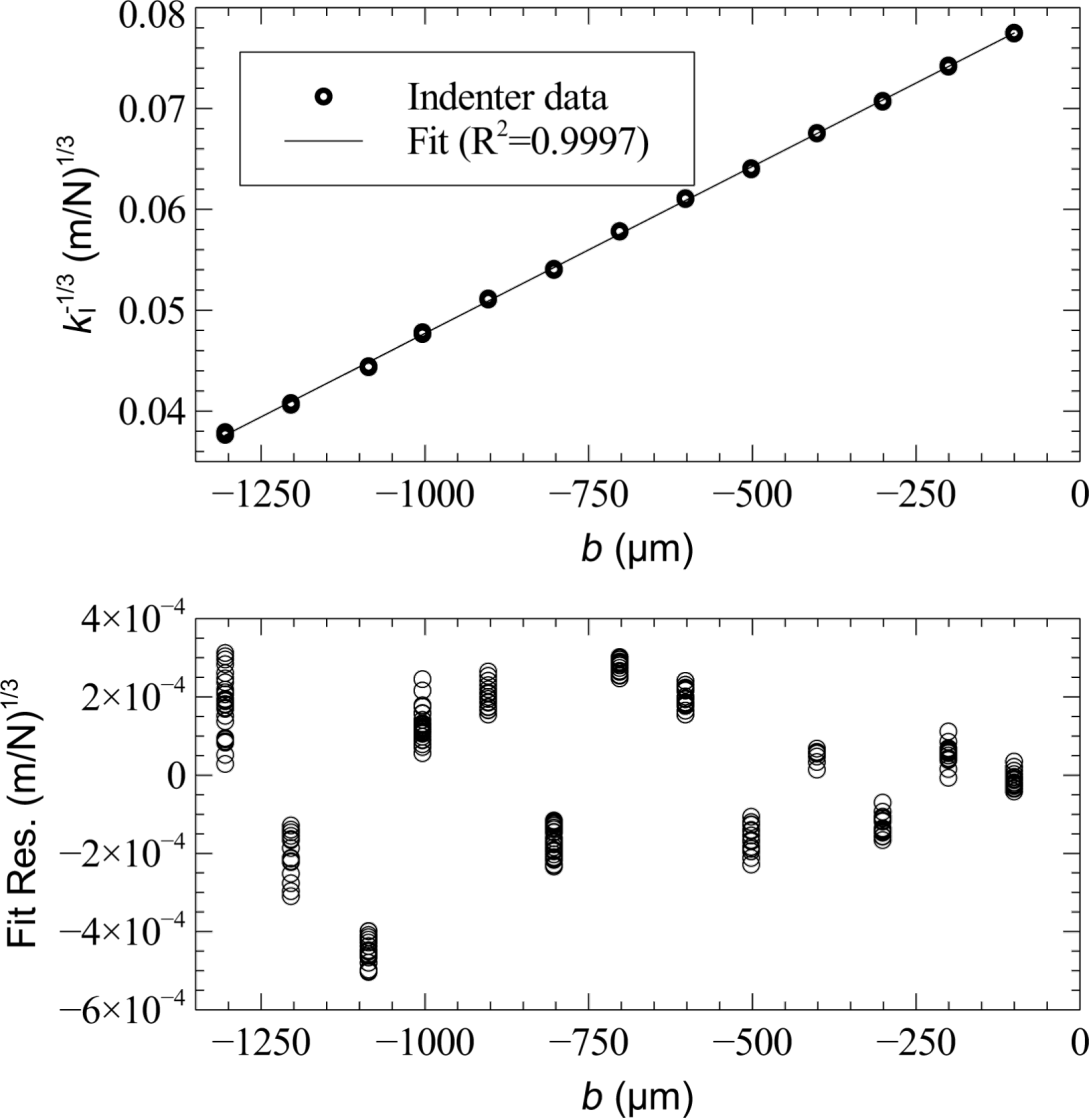
Nanoindenter measurements from a tunning fork tine. 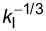 vs *b* is plotted where *k*_I_ is the spring constant measured at an offset *b* from the distal edge of the tine. A linear least-squares regression determines *EI* and *L* of the tine.

Figure 7 shows the indentation measurements for a single sensor (qPlus A). Plotting 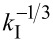 vs *b* reveals excellent linearity as indicated by the coefficient of determination *R*^2^ = 0.9997. The goodness of fit and uncorrelated residuals serve to validate the use of Euler–Bernoulli beam theory to model the flexural mechanics of the tine. A least-squares regression to indentation data determines the flexural rigidity and effective cantilever length.

The effective length, flexural rigidity, and nominal spring constant for several sensors are provided in Table 2. The combined standard uncertainty in the parameters includes Type A (statistical) uncertainty, which we estimate from the regression, and Type B (non-statistical) uncertainty, which includes (i) 1% uncertainty in the calibration of both force and indention of the nanoindenter and (ii) ±5 μm uncertainty in the positioning the indenter tip with respect to the distal edge of the tine. The details of the analysis are provided in the Appendix.

**Table 2:** Mechanical characterization of qPlus sensors with nanoindentation.

sensor	mounting	*k_z_* (*B* = 0) (N/m)	*L*_eff_ (μm)	*EI (*μN·m^2^)

qPlus A	tine	1897 ± 29	2442 ± 16	9.20 ± 0.13
qPlus B	tine	1912 ± 29	2440 ± 17	9.27 ± 0.14
qPlus C	tine	1912 ± 29	2470 ± 18	9.60 ± 0.16
qPlus D	tine	1888 ± 29	2419 ± 17	8.91 ± 0.14
qPlus E	base	2143 ± 33	2339 ± 17	9.14 ± 0.15
qPlus F	base	2153 ± 34	2327 ± 19	9.04 ± 0.18

Figure 6 shows the spring constant *k_z_* vs the effective length with a color bar for the flexural rigidity. There are two distinct values of the effective length directly corresponding to the two mounting configurations. The additional constraint provided by the glue at the base effectively shortens the tine leading to a stiffer spring constant. The flexural rigidity of the tine, however, is unaffected by the mounting configuration, as expected.

Assuming the reported values for the effective length and spring constant at zero tip offset, it is possible to determine the spring constant of the qPlus sensor from only the plane-view geometry and infer *k_z_*(*B*) simply by

[13]
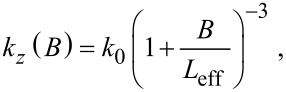


where *k*_0_ = *k_z_*(0) is the spring constant at zero offset. We estimate from Equation 13 that the spring constant of the qPlus sensor can be determined with less than 2% relative standard uncertainty for moderate tip offsets (less than ±100 μm with this method, see Appendix section).

Finally, the agreement between experiment and theory suggests that the spring constant of the tuning fork can be predicted reasonably well from the geometry and Young’s modulus of the tine, being careful to include the contribution of the peaked ridge. The cross-section geometry can be determined by cleaving a tine as shown in Figure 1. Adopting this approach for the E158 tuning fork, we predict *EI* = 9.20 × 10^−6^ N·m^2^ and *k_z_* = 2000 ± 130 N/m, where the dominant uncertainty is an estimated ±50 μm in the effective length due to the non-ideal boundary condition. This approach could be used for tuning forks other than the E158 with an estimated uncertainty of 10%.

## Discussion

Because of their development for consumer timing applications, we can expect very little variation in the mechanical properties of a given commercial tuning fork. Some additional variables are introduced, however, by the attachment of a tip and the mounting of the sensor. Provided the tip height is sufficiently small (approximately less than 400 μm) and the mounting of the sensor is consistent, the additional variables can be determined from the plane-view geometry. We estimate that the nominal spring constant of the E158 qPlus sensor is 1902 ± 29 N/m, with an effective length of 2443 ± 21 μm. This spring constant, however, can be significantly higher if the base of the tuning fork is also constrained by the glue, also reducing the effective length of the beam. We note that while this value is significantly higher than the commonly-assumed spring constant of 1800 N/m [1], an estimate that gets significantly worse if the tip is inset from the end of the beam; the estimate is accurate for tips offset by about 50 μm from the end of the tine. Our values are not consistent with the range of 1480–1708 N/m estimated by Falter et al. [29]. We expect that complications from the gluing of tips between the tuning fork tine and load cell may have contributed to the poor agreement between theory and experiment found in [29]. On the other hand, the nanoindentation experiments presented here are highly reproducible and demonstrate excellent agreement with the theoretical model. There is, however, some potential for further work examining the effect of the mounting on the qPlus spring constant. We have observed a small variation (less than 3%) in stiffness by testing at a lateral offset from the beam axis.

Finally, we note that for sufficiently long tips, the compliance of the tip contributes to the parasitic tip motion [32–33]. This, in turn, influences the spring constant and force spectroscopy results presented here. To quantify the effect of the tip compliance, we construct a finite element model corresponding to our beam model and 50 μm diameter tungsten wire tip. The tip was first modelled as a rigid domain; the inertial loading of the beam gives results consistent with our analytical model. The rigid constraint was then removed, allowing the tungsten tip to deform elastically. Comparing the results for elastic and rigid tip models, we find the effect of tip compliance is negligible for tip heights below 400 μm, contributing about 1% to total parasitic tip motion and less than 0.05% to *k_z_*. This effect becomes more pronounced for tips exceeding 750 μm, contributing over 25% to the overall parasitic tip motion and over 1% to *k_z_*. As such, the use of shorter tips allows the parasitic tip motion to be related to the bending of the tine with negligible contribution from tip compliance.

## Conclusion

In summary, we have developed a mathematically rigorous model of the qPlus sensor that includes the effect of finite tip lengths on the reconstructed tip–sample force in ncAFM. A grid spectroscopy simulation with a Morse potential shows that significant errors in the force reconstruction occur for tip heights greater than 400 μm. A quasi-static nanoindentation method is used to validate Bernoulli–Euler beam theory with fixed-free boundary conditions for modeling the flexure of the tuning fork tine. Indentation data provides the effective length, flexural rigidity, and nominal spring constant of the tine with an estimated uncertainty of 2%. Finally, we have proposed two methods for estimating the spring constants of qPlus sensors with finite tips. The first is to extrapolate the nominal value provided for the E158 for a given measured tip offset. This method has an estimated uncertainty in the neighborhood of 2%. The second is simply to estimate the spring constant from the Young’s modulus and geometry of the tuning fork, taking care to measure the dimensions of the cross section. We estimate the uncertainty in this method is closer to 10%, which comes primarily from limited knowledge of the effective cantilever length.

## Appendix

### Discretization of the Euler–Bernoulli Equation

The process by which the partial differential equation governing the continuous tuning fork tine is reduced into a set of ordinary differential equations is referred to as discretization and is briefly described here. The process involves first solving for the normal eigenmodes of the cantilever for the case of *V* = 0 and second, using the unperturbed normal modes as a basis for the discretization.

The normal modes of the cantilever are determined by substituting *V* = 0 and *w*(*X*,*t*) = Φ(*X*)exp(*i*ω*t*) into Equation 3. The nontrivial solutions correspond to the roots of the characteristic equation

[14]
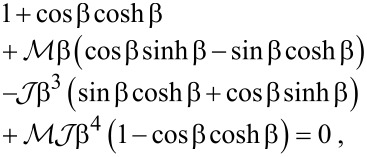


where 
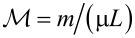
 and 
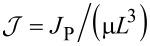
, and the dispersion relation is

[15]
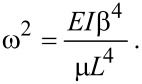


The eigenfunctions are given by

[16]
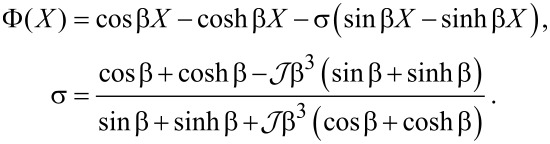


There are countably-infinite solutions to Equation 14, which form an admissible basis for a Galerkin discretization [38] of Equation 3. Equation 5 follows from a single-term truncation.

### Uncertainty Estimates

In this section we provide an analysis of the uncertainty in indentation measurements. The nanoindenter measures force *F*_I_ vs indentation δ at a specified displacement for the distal edge of the tuning fork tine. Let 
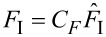
 and 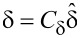 where *C_F_* and *C*_δ_ are calibration constants, both of which are estimated to have 1% relative standard uncertainties, denoted 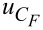 and 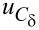, respectively. Finally, we estimate a ±5 μm uncertainty in the positioning of the indenter tip with respect to the distal edge, which we denote *U*_E_. Relative uncertainty estimates are summarised in Table 3.

**Table 3:** Relative uncertainty contributions. Type A estimates correspond to the indentation data from the qPlus A sensor.

source	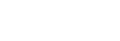	*u_EI_*	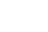

indenter calibration	0.014	0.014	0.0067
distal edge position	0.0061	0	0.0020
type A	0.0011	0.0031	0.0011

Let *u* refer to the standard relative uncertainty of a specified parameter. The relative standard uncertainty 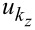 in *k_z_* is estimated by

[17]
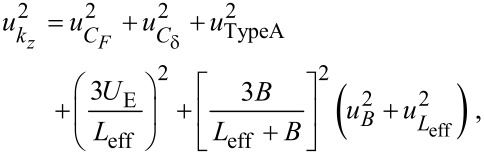


where 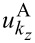 represents the type-A (statistical) uncertainty estimated from the regression. The uncertainty in the flexural rigidity is estimated by

[18]
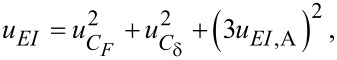


where, *u_EI_*_,A_ represents the type-A uncertainty in *EI*, and the uncertainty in the effective length is estimated by

[19]